# Biologically Individualized Radiotherapy Based on PET: A Novel Approach to Treatment Optimization of Head and Neck Cancer

**DOI:** 10.2967/jnumed.125.270403

**Published:** 2026-03

**Authors:** Marta Lazzeroni, Ana Ureba, Henning Schäfer, Nils H. Nicolay, Alexander Rühle, Dimos Baltas, Alexandru Dasu, Philipp T. Meyer, Michael Mix, Iuliana Toma-Dasu, Anca L. Grosu

**Affiliations:** 1 Department of Physics, Stockholm University, Stockholm, Sweden;; 2 Department of Oncology and Pathology, Karolinska Institute, Stockholm, Sweden;; 3 Department of Radiation Oncology, Medical Center, Medical Faculty Freiburg, German Cancer Consortium Partner Site Freiburg, Freiburg,Germany;; 4 Department of Radiation Oncology, University of Leipzig Medical Center, Leipzig, Germany;; 5 Skandion Clinic, Uppsala, Sweden;; 6 Department of Immunology, Genetics and Pathology, Uppsala University, Uppsala, Sweden; and; 7 Department of Nuclear Medicine, Faculty of Medicine, Medical Center–University of Freiburg, University of Freiburg, Freiburg, Germany

**Keywords:** dual-tracer PET, head and neck squamous cell carcinoma, biologically individualized radiotherapy, tumor hypoxia, clonogenic cell density

## Abstract

Current radiotherapy for malignant tumors often adopts a “one-size-fits-all” approach, prescribing the same irradiation dose for patients with similar clinical indications. However, advancements in functional imaging allow for biologically individualized strategies, with dose distribution tailored to the specific tumor biology. This study proposes a novel approach to biologically individualized radiotherapy, exploiting the synergistic combination of the tumor clonogenic cell information from [^18^F]FDG PET images and radiosensitivity from [^18^F]fluoromisonidazole (FMISO) PET images. **Methods:** Twenty-eight patients with head and neck squamous cell carcinoma (HNSCC) were analyzed. Using imaging biomarkers, individualized tumor profiles were obtained from oxygen partial pressure and clonogenic cell density maps derived from [^18^F]FMISO and [^18^F]FDG PET, respectively. Dose-escalated radiotherapy plans aiming at 95% tumor control probability (TCP) were generated using automated planning. Plans were assessed for clinical feasibility and expected TCP. **Results:** Planned dose distributions achieved greater than 90% TCP in all cases. All treatment plans met standard clinical feasibility criteria for the main organs-at-risk constraints, except for the few cases with significant target overlap, demonstrating the overall feasibility of the personalized strategy. **Conclusion:** The proposed biologically individualized treatment strategy demonstrated feasibility and clinical applicability. Combining [^18^F]FDG and [^18^F]FMISO PET imaging potentially shifts the success rate of HNSCC treatment from approximately 60% at 5 y, as reported in the literature, to a projected TCP of 90%. This treatment strategy holds promise for improving patient outcomes through more precise and effective treatment.

## Visual Abstract

**Figure:**
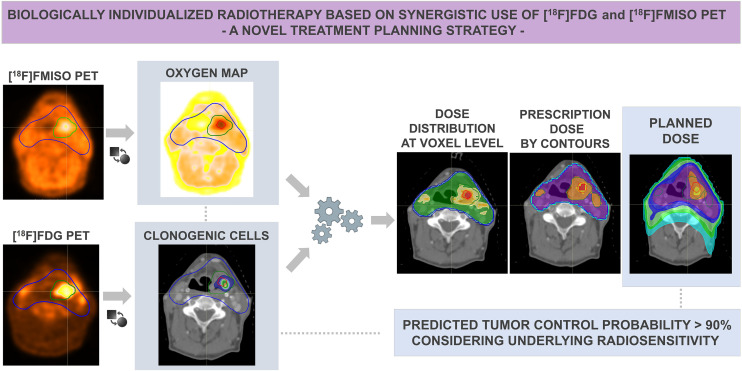

Advances in functional imaging are paving the way for biologically individualized radiotherapy, accounting for the most critical radiobiologic factors influencing treatment response: tumor cellularity and hypoxia (1). This marks a shift from conventional radiotherapy, which largely relies on empiric dose prescription and physical dose distribution optimization on the basis of tumor type and normal tissue anatomy. Standard practice focuses on delivering a uniform dose to the target while sparing healthy tissues and fulfilling the organ-at-risk (OAR) constraints. However, intrinsic radiosensitivity and spatiotemporal variations in radioresistance, both critical to treatment outcomes, are often overlooked (1–3).

Biologic heterogeneity in head and neck squamous cell carcinoma (HNSCC), manifesting as interpatient and intrapatient variations, contributes to recurrence and treatment failure (3). Factors such as genetic diversity, metabolic rates, cellular density, and hypoxia lead to variable radiosensitivity within the same tumor (4). Growing evidence identifies these variations as key drivers of recurrence, reinforcing the need for personalized treatment. When relevant radiobiologic targets are identified, dose escalation to selected subvolumes, while sparing OARs, may improve local control (5).

Functional imaging, particularly PET, has advanced our understanding of tumor biology, enabling more precise treatment individualization. Several dose-painting strategies based on PET hypoxia imaging have been proposed, from empiric methods to radiobiologic modeling (6). These include simple linear conversions of tracer uptake into absorbed dose (7), as well as advanced models that compute dose distributions required to reach the desired tumor control probability (TCP) (6). For example, [^18^F]fluoromisonidazole (FMISO) PET enables derivation of oxygen partial pressure (pO_2_) maps to guide radiosensitivity-based dose modification (6). Although these techniques show promise in silico and for clinical feasibility (6), combining information from multiple PET tracers for dose prescription remains largely unexplored.

In this work, we explored a biologically guided dose-painting strategy for HNSCC, using [^18^F]FDG PET to estimate clonogenic cell density and [^18^F]FMISO PET to quantify hypoxia-related radioresistance. These biologic maps informed heterogeneous dose prescriptions, with escalated doses delivered to subvolumes with unfavorable characteristics while maintaining clinically acceptable OAR sparing. By tailoring dose distribution to both tumor cellularity and oxygenation, this approach aims to enhance tumor control and improve patient outcomes.

## MATERIALS AND METHODS

### Patient Dataset and PET/CT Images

This study included 28 patients with advanced HNSCC who were enrolled in a prospective, monocentric, noninterventional [^18^F]FMISO trial (DRKS00003830), approved by the ethics committee of the University of Freiburg (Ref. 479/12). All procedures complied with the Declaration of Helsinki, and patients provided written informed consent.

**FIGURE 1.**
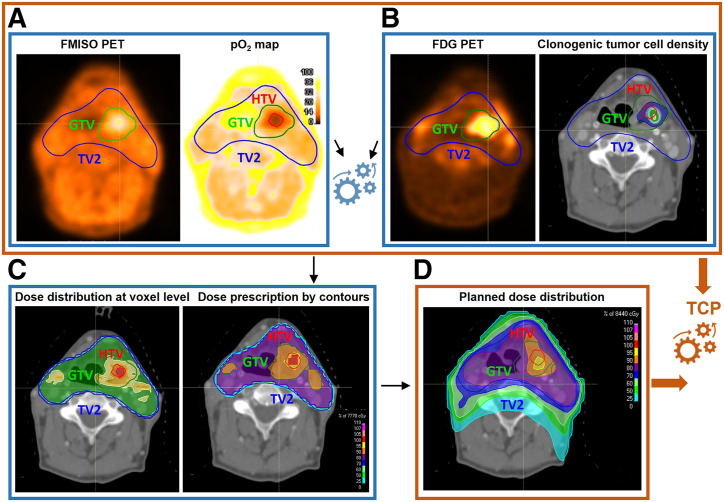
Study workflow. (A) [^18^F]FMISO PET was used to derive voxel-level pO_2_ maps. (B) [^18^F]FDG PET provided information on clonogenic tumor cell distribution. These datasets informed voxel-level dose prescription to counteract radioresistance, determining required dose escalation for hypoxic volume (C). Resulting planned dose distribution (D), together with radiosensitivity and clonogenic cell density maps, was used to predict TCP. Color scale in pO_2_ map in panel A shows oxygen distribution (range, 0–100 mm Hg), whereas color scales in panels C and D show percentage of maximum dose in treatment plans.

**FIGURE 2.**
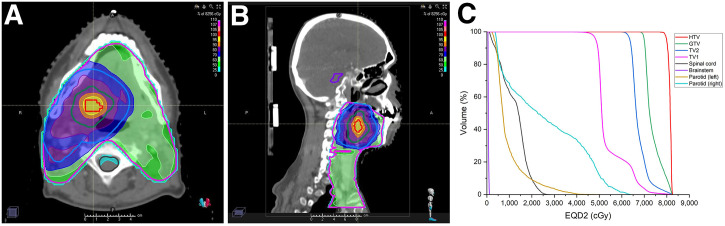
Illustration of planned dose distribution for representative patient. Transversal (A) and sagittal (B) planes show dose distribution targeting dose escalation in hypoxic compartment. (C) Corresponding dose-volume histograms for key structures, expressed as EQD2. Color scales in panels A and B depict percentage of maximum dose in treatment plan.

Patients received definitive concomitant cisplatin-based chemoradiotherapy at the University Medical Center Freiburg. Gross tumor volume (GTV), including primary tumor and pathologic lymph nodes, was delineated via CT, MRI, and [^18^F]FDG PET (with 40% of the maximum SUV threshold). Patients were prescribed 50 Gy (2 Gy/fraction) in target volume (TV) 1, followed by a 10-Gy boost in TV2, or 5 Gy in TV2 plus 5 Gy in TV3 (Figs. 1 and 2).

Pretreatment [^18^F]FMISO and [^18^F]FDG PET/CT scans were performed at −5 ± 3 and −9 ± 6 d, respectively. [^18^F]FDG PET/CT images were available for all but 3 patients and were acquired approximately 100 min after injection (5.0 ± 0.5 MBq/kg). [^18^F]FMISO PET acquisition began roughly 160 min after injection (302 ± 18 MBq), covering the head and neck region in radiotherapy position using a thermoplastic mask. Scan times were 10 min for [^18^F]FDG PET and 20 min for [^18^F]FMISO PET. Images were reconstructed at 2 × 2 × 2 mm^3^ voxel size using a line-of-response based ordered-subset iterative time-of-flight algorithm using spheric coordinates with 3 iterations, 33 subsets, and a relaxation parameter of 0.35 for smoothing. Images were corrected for randoms, scatter, and attenuation via CT.

Both PET scans were imported into the treatment planning system (RayStation; RaySearch Laboratories) and rigidly registered to the planning CT (CT_plan_) using bony anatomy.

Baseline demographics and clinical outcomes are summarized in Table 1. The median age at treatment start was 60.5 y, and most patients were male (93%). Six patients (21%) had human papilloma virus–positive tumors. During follow-up, 16 patients (57%) had died, 15 (53%) experienced locoregional recurrence, 9 (32%) had distant failure, and 21 (75%) had disease progression.

**TABLE 1.** Patient Demographics and Treatment Outcomes (*n* = 28)

Patient no.	Age (y)	Sex	HPV status*	Tumor site	OS	LRR	DF	PFS
1	60	M	1	Orohypopharynx	0	0	0	0
2	53	M	0	Oropharynx	1	1	1	1
3	54	M	0	Hypopharynx	1	1	0	1
4	52	M	1	Oropharynx	0	0	0	0
5	61	M	0	Hypopharynx	1	1	1	1
6	54	M	1	Hypopharynx	0	1	0	1
7	66	M	0	Oropharynx	1	1	1	1
8	51	M	0	Orohypopharynx	0	1	0	1
9	61	M	1	Oropharynx	0	0	0	0
10	70	M	1	Oropharynx	0	0	0	0
11	61	M	0	Oropharynx	1	1	0	1
12	60	M	1	Oropharynx	0	0	0	0
13	49	M	0	Hypopharynx	1	0	0	1
14	60	M	0	Hypopharynx	1	1	0	1
15	63	M	0	Oropharynx	1	1	1	1
16	64	F	0	Oropharynx	1	0	1	1
17	41	M	0	Oropharynx	1	1	1	1
18	54	F	0	Orohypopharynx	1	0	0	1
19	69	M	0	Orohypopharynx	1	1	0	1
20	34	M	0	Larynx	0	0	0	0
21	67	M	0	Hypopharynx	1	0	1	1
22	42	M	0	Larynx	0	1	0	1
23	61	M	0	Orohypopharynx	1	1	1	1
24	67	M	0	Larynx	0	0	0	0
25	57	M	0	Larynx	0	1	0	1
26	57	M	0	Oropharynx	0	1	0	1
27	67	M	0	Oral cavity	1	0	0	1
28	65	M	0	Oropharynx	1	0	1	1

* Values coded as 1 (event present/positive status) or 0 (event absent/negative status).

HPV = human papillomavirus; OS = overall survival; LRR = locoregional recurrence; DF = distant failure; PFS = progression-free survival.

### Personalized Dose-Escalation Workflow

Figure 1 illustrates the biologically individualized radiotherapy dose-escalation workflow used in this study. The subsequent subsections describe each step of the process.

For clarity, a glossary of radiotherapy-specific terms is provided in Supplemental Table 1 (available at http://jnm.snmjournals.org).

#### Dose Prescription Based on Tumor Oxygenation and Cell Density

[^18^F]FMISO PET uptake was converted into a pO_2_ distribution by applying a nonlinear sigmoid conversion function (Eq. 1) at the voxel level, as detailed by Toma-Dasu et al. (6):

$$pO_{2}=\frac{c(a-{\mathrm{Uptake}}_{\mathrm{FMISO}}\left(r\right))}{b\;+\;{\mathrm{Uptake}}_{\mathrm{FMISO}}\left(r\right)-a},$$ Eq. 1

where *a*, *b*, *c* were [^18^F]FMISO reaction-specific parameters equal to 10.9, 10.7, and 2.5 mm Hg, respectively. ${\mathrm{Uptake}}_{\mathrm{FMISO}}$ was calculated by dividing the voxel values in the [^18^F]FMISO PET images by the average value in a well-oxygenated volume, and the results were multiplied by the tracer uptake predicted by the conversion function for the assigned pO_2_ in the well-oxygenated volume. The deep neck muscle volume, delineated by an expert radiologist, was chosen as the well-oxygenated volume with an assigned pO_2_ of 30 mm Hg (8). The hypoxic target volume (HTV) was defined within TV2 by thresholding pO_2_ maps at 10 mm Hg.

Clonogenic cell density ($\rho_{N})$ was estimated from [^18^F]FDG uptake using a linear function (9,10):

$$\rho_{N}=A\cdot \left({\mathrm{Uptake}}_{\mathrm{FDG}}-1\right).$$ Eq. 2

Here, ${\mathrm{Uptake}}_{\mathrm{FDG}}$ was the [^18^F]FDG signal normalized to the deep neck muscle. Slope *A* was calibrated so that maximum normalized uptake (∼30) in TV2 across the patient dataset mapped to a maximum cell density carrying capacity ($\rho_{N\max}=10^{9}\;$cm^−3^) (11). For patients lacking [^18^F]FDG PET, a uniform cell count of 10^7^ cells cm^−3^ was assigned to TV2 (6).

Toma-Dasu et al. (6) also proposed a method to compute voxelwise dose distributions to achieve a specified TCP, accounting for heterogeneity in both cell density and radiosensitivity. On the basis of this distribution, a corresponding dose-escalation strategy can be formulated to achieve the desired level of TCP by prescribing a homogeneous dose across different subtargets within the TV2 in a predefined number of fractions (i.e., dose painting by contours). The subtargets TV2-GTV (TV2 excluding GTV), GTV-HTV (GTV excluding HTV), and HTV were defined via volume algebra in RayStation. This approach was implemented using the following equation:

$$D_{P}=\frac{\bar{D}}{1-\frac{\gamma }{2T\mathrm{CP}}{\left(\frac{\sigma_{D}}{\bar{D}}\right)}^{2}},$$ Eq. 3

where γ is the slope of the TCP curve (set to 4), $\bar{D}$ is the average dose in the volume of interest (VOI), and σ*_D_* is the SD of the dose within the subtargets (6).

#### Automated Volumetric Modulated Arc Therapy Planning with Integrated Boost: Dosimetric and Radiobiologic Evaluation

Volumetric modulated arc therapy plans using two 6-MV photon arcs were generated in RayStation. An in-house automated pipeline (12) applied the same objective functions and weights across patients. No manual adjustments were made, except for a few cases with suboptimal TCP. Dose distributions were calculated using robust optimization with ±3 mm setup uncertainties and were delivered in 35 fractions with an integrated boost.

Plans were evaluated for target coverage and OAR constraints, expressed as equivalent dose in 2 Gy (EQD2). The conformity index (13) and homogeneity index (13) were calculated for TV2-GTV, GTV-HTV, and HTV. OAR constraints were set as follows: near-maximum dose (the dose received by the hottest 2% of the organ volume, *D*_2%_) of less than 45 Gy for the spinal cord and brainstem, *D*_2%_ of less than 70 Gy and $\bar{D}$ of less than 54 Gy for the mandible, and $\bar{D}$ of less than 20 Gy for at least 1 parotid gland, or $\bar{D}$ of less than 25 for both parotid glands combined.

The prescribed dose for achieving a specified level of TCP was calculated by considering radiosensitivity and cell density. A radiobiologic evaluation of the treatment plan was conducted to determine whether the optimized dose distribution could achieve the desired target control. The TCP in the VOI (${\mathrm{TCP}}_{\mathrm{VOI}})$ was assessed using the dose distribution in the nominal plan $D\left(i\right)$, the radiosensitivity maps derived from the [^18^F]FMISO PET scan, and the cell density from the [^18^F]FDG PET:

$${\mathrm{TCP}}_{\mathrm{VOI}}=\prod_{i}^{N_{\mathrm{tot},\mathrm{VOI}}}\text{TCP}\left(i\right)=\prod_{i}^{N_{\mathrm{tot},\mathrm{VOI}}}\text{exp}\left(-\eta (i)\mathrm{SF}(i)\right),$$ Eq. 4

where η(i) is the initial number of cells per voxel of volume *v* and given by $v\rho_{N}\left(i\right)$. The surviving fraction of cells is

$$\mathrm{SF}\left(i\right)=\exp\left[-\left(\frac{\alpha }{f\left(i\right)}D\left(i\right)+\frac{\beta }{f^{2}(i)}\frac{D^{2}\left(i\right)}{n}\right)\right].$$ Eq. 5

The term *f* represents a voxel distribution of dose modification factors derived from pO_2_ maps as

$$f\left(i\right)=\frac{{\mathrm{OER}}_{\max}\left(k+{\mathrm{pO}}_{2}(i)\right)}{{k+\mathrm{OER}}_{\max}\cdot {\mathrm{pO}}_{2}(i)},$$ Eq. 6

with a maximum oxygen enhancement ratio (OER_max_) of 3 and a *k* of 2.5 mm Hg (6).

Automated planning was implemented exclusively for the biologically guided plans in this study to ensure consistency across patients. In contrast, the clinical and delivered treatment plans were generated using standard institutional workflows, which did not use automated optimization.

## RESULTS

Tumor hypoxia (pO_2_ ≤ 10 mm Hg) was confirmed in 18 of 28 patients; 8 had an HTV of less than 1 cm^3^.

Figure 2 shows an example treatment plan with dose escalation in the HTV, showing transversal and sagittal planes, and EQD2-based dose-volume histograms for the main radiotherapy targets and OARs.

**FIGURE 3.**
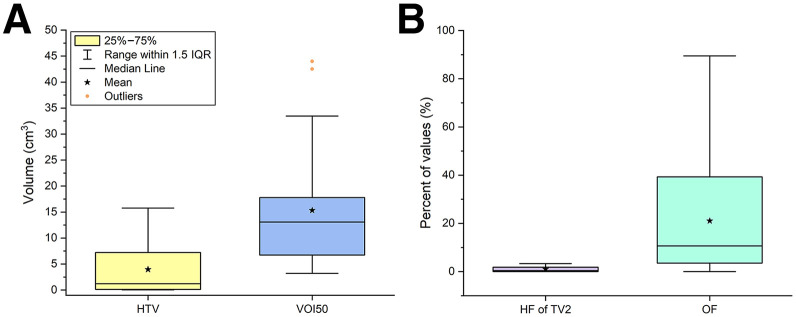
Distribution of characteristics of hypoxic compartments and [^18^F]FDG PET avid regions. (A) Box plots HTV (defined as pO_2_ ≤ 10 mm Hg) and [^18^F]FDG PET–avid volume (VOI50). (B) Box plots illustrating hypoxic fraction (defined as ratio between HTV and radiotherapy volume TV2) and overlap fraction (OF; intersection of HTV and VOI50, expressed as percentage of VOI50). These plots demonstrate that HTVs were generally smaller than [^18^F]FDG PET–avid regions and that overlap between hypoxic and metabolically active regions was only partial, underscoring complementary nature of [^18^F]FMISO and [^18^F]FDG PET in characterizing tumor biology.

Figure 3 shows the distribution of the characteristics of the hypoxic compartments and [^18^F]FDG–avid regions in the investigated patient cohort. The hypoxic compartment was characterized by its volume and hypoxic fraction with respect to the TV2. The region of increased [^18^F]FDG uptake was characterized by the volume of the region corresponding to 50% of the maximum SUV (VOI50). The overlap fraction between HTV and VOI50 was also considered in the analysis, calculated as the intersection between the HTV and VOI50 and expressed as a percentage of the VOI50. Detailed information for each patient is provided in Supplemental Table 2.

Automated planning achieved high-quality treatment plans for all patients without manual intervention, ensuring reproducibility of the dose-escalation strategy. Supplemental Table 3 presents the conformity and homogeneity indices for values from automated planning, without manual postprocessing, whereas corresponding box plot results are presented in Supplemental Figure 1.

**FIGURE 4.**
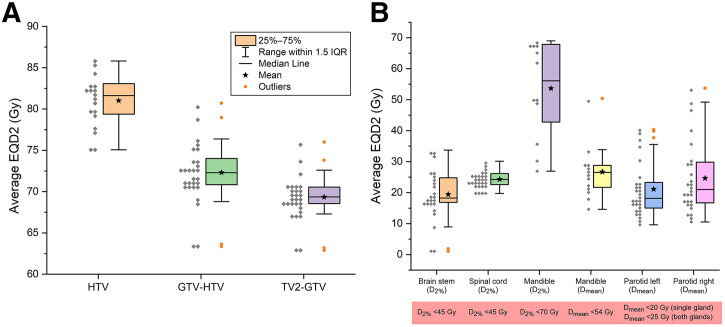
Dosimetric characteristics of treatment plans. Box plots of average EQD2 to (A) target volumes (HTV, GTV-HTV, TV2-GTV) and (B) main organs at risk (parotid glands, spinal cord, brainstem, mandible). Individual patient data points are represented as gray diamonds. This figure highlights achieved balance between escalation to hypoxic subvolumes and sparing of OARs. D_2%_ = dose received by the hottest 2% of the organ volume; D_mean_ = mean dose.

Figure 4 shows box plots of the dosimetric characteristics of the plans. The average EQD2s were 81 ± 3 Gy (HTV), 72 ± 4 Gy (GTV-HTV), and 69 ± 3 Gy (TV2-GTV). All 28 plans met clinical constraints for the brainstem, spinal cord, and mandible. Parotid glands were spared in 75% of the cases (21/28); in other cases, glands were located within the TV1, and target coverage was prioritized. Detailed information for each of the patients is given in Table 2.

**TABLE 2.** Dosimetric Evaluation of Individual Treatment Plans

	Average EQD2						EQD2_2%_		
Patient no.	HTV	GTV-HTV	TV2-GTV	Mandible	Parotid left	Parotid right	Brainstem	Spinal cord	Mandible
1	83.2	76.4	70.4	28.8	17.9	53.7	26.2	28.7	69
2	82.4	74	70.9	28.2	21.4*	35.6*	25.2	26.8	65.5
3	/	71.2	68.5	NA	23.7	24.1	NA	23.9	NA
4	/	68.8	67.3	NA	21.7	13.3	18.7	23.5	NA
5	/	71.8	69.8	NA	14.3	29.5	13.9	20.1	NA
6	83.1	73	69.6	NA	22.7	19.9	17.5	20.8	NA
7	/	71.2	68.7	33.9	21.6*	40.5*	22.8	25.2	67.9
8	82.4	72.8	70.7	27.2	35.6*	29.8*	17.3	25.4	61.9
9	80.7	75.8	70.8	NA	40.3*	21*	17.2	26.2	NA
10	77.2	72.8	70.1	NA	25.9*	30.4*	24.4	22.7	NA
11	79.8	72.9	69.9	NA	17.3	19.4	23.1	19.8	NA
12	81.4	73.2	67.6	NA	9.6	26.2	1.1	23.8	NA
13	/	69.3	67.4	NA	17.1	21.6	18.5	24.6	NA
14	/	70.5	68.7	NA	15.2	16.1	NA	22.6	NA
15	79.4	72.9	70.2	NA	12.9	23.2	20.2	24.8	NA
16	82.7	75.2	69.5	NA	14.9	17.6	14.4	20.1	NA
17	81.9	74	70.7	NA	37.7	12.8	33.8	27.4	NA
18	84.5	75.3	72.6	NA	20.2	15.6	33.5	23.4	NA
19	/	70.9	68.8	26.5	17.7	18	1.9	24.7	67.9
20	/	63.6	63.2	18.9	10.8	10.5	16.9	21.9	30.7
21	85.8	79	73.8	21.7	12.7	12.4	18	22.6	36.3
22	/	63.4	62.9	14.6	17.2	17.3	17.9	23.7	26.9
23	75.1	70.7	69	24.5	18.4	16.7	NA	26.1	49.8
24	75.1	71.2	67.3	23.2	16.9	NA	NA	27.1	50.3
25	77.8	70.7	68.6	20.6	13.5	20.3	9	25.2	49.3
26	80	71.8	69.1	50.4	30.8*	49.2*	16.7	26.3	68.3
27	/	71.3	69.2	NA	22.9	23	26.4	23	NA
28	85.7	80.7	76	NA	39.8*	47.5*	31.6	30.2	NA
Median	81.6 (75.1–85.8)	72.3 (63.4–80.7)	69.4 (62.9–76.0)	25.5 (14.6–50.4)	18.2 (9.6–40.3)	21.0 (10.5–53.7)	18.3 (1.1–33.8)	24.2 (19.8–30.2)	56.1 (26.9–69.0)
Mean ± SD	81.0 ± 3.2	72.3 ± 3.7	69.3 ± 2.6	26.5 ± 9.1	21.1 ± 8.6	24.6 ± 11.6	19.4 ± 8.3	24.3 ± 2.6	53.6 ± 15.5

* OAR EQD2 constraint not met.

/ = nonhypoxic; NA = structure delineation not available.

Radiobiologic evaluation results for TV2-GTV, GTV-HTV, HTV, and whole TV2 are shown in Table 3. Predicted TCP, accounting for the planned dose distribution, image-derived radiosensitivity, and clonogenic cell maps, was 90% or greater for all HTV and GTV-HTV cases. For TV2-GTV and TV2, 86% (24/28) and 79% (22/28) of cases, respectively, had a TCP of 90% or greater. Among the 6 TV2 cases with a TCP of less than 90%, 3 had values of at least 88%; the remaining 3 had substantially lower TCP (0%, 0%, 59%) because of failures in TV2-GTV. These plans were retrospectively adjusted, yielding a TCP exceeding 90% in all cases (Table 3). For the 3 patients without [^18^F]FDG PET images, a uniform clonogenic cell density was assumed. Their predicted TCP values were within the overall range of the cohort, with no systematic deviation observed. Supplemental Figure 2 shows box plots of the TCP results.

**TABLE 3.** Radiobiologic Evaluation of Planned Dose Distributions

	Predicted TCP			
Patient no.	TV2-GTV	GTV-HTV	HTV	TV2
1	0 (95*)	99 (99*)	100 (100*)	0 (94*)
2	94	99	100	93
3	92	99	100	91
4	97	98	100	95
5	90 (94*)	100 (100*)	100 (100*)	89 (93*)
6	93	100	100	93
7	89 (95*)	99 (99*)	100 (100*)	88 (94*)
8	93	99	100	91
9	96	100	100	95
10	95	99	100	94
11	96	99	100	95
12	96	99	100	96
13	96	99	100	95
14	95	99	100	94
15	96	99	100	95
16	93	100	100	93
17	93	99	100	92
18	0 (95*)	93 (99*)	99 (99*)	0 (93*)
19	90 (95*)	99 (99*)	100 (100*)	89 (94*)
20	96	99	100	96
21	95	100	100	95
22	96	100	100	96
23	97	99	100	96
24	95	100	100	95
25	96	99	100	95
26	61 (93*)	96 (99*)	100 (100*)	59 (92*)
27	95	99	100	94
28	91	99	100	90
Median	95 (95*)	99 (99*)	100 (100*)	94 (94*)
Mean ± SD	86 (95*) ± 25 (2*)	99 (99*) ± 1 (0*)	100 (100*) ± 0 (0*)	86 (94*) ± 25 (2*)

* TCP values in parentheses were obtained after manual plan refinement.

TCP was calculated retrospectively based on the planned dose, incorporating clonogenic cell counts from [^18^F]FDG PET and radioresistance data from [^18^F]FMISO PET.

Data expressed as a percentage.

## DISCUSSION

We developed an individualized, biologically guided dose-escalation strategy targeting 2 key radiation resistance parameters: tumor cellularity and tumor hypoxia, derived from [^18^F]FDG and [^18^F]FMISO PET, respectively. This multitracer PET–based approach, tested in 28 patients with HNSCC, proved clinically feasible from dosimetric and radiobiologic perspectives. Boosting the HTV to 75–86 Gy EQD2 (mean, 81 Gy) while respecting OAR constraints resulted in a TCP of greater than 90%, a notable improvement over the ∼60% reported in the literature.

Our method builds on an established model that computes dose maps to counteract hypoxia-related radioresistance for a given TCP (6). TCP depends on clonogenic cell count, influenced by tumor volume and cell density. In our approach, [^18^F]FDG uptake estimates voxelwise cell density. Although a correlation between [^18^F]FDG uptake and cell density has been hypothesized (3,9,14–19), its exact nature remains uncertain. Nonetheless, comparisons between apparent diffusion coefficient maps and [^18^F]FDG PET in HNSCC patients have shown associations between apparent diffusion coefficient–derived entropy and both metabolic tumor volume and total lesion glycolysis (16). Another study reported an inverse correlation between SUV_max_ and the minimum apparent diffusion coefficient in HNSCC (17). In the absence of a validated conversion model, we pragmatically adopted a linear relationship between normalized tracer uptake and cell density, ranging from a baseline density of 10^7^ cm^−3^ to a carrying capacity of 10^9^ cm^−3^ (11). The resulting EQD2 values averaged 69.3 ± 2.6 Gy in the rim TV2-GTV. These values are consistent with standard clinical prescriptions for the clinical target volume in similar tumor indications (20), supporting the plausibility of our model.

For 3 patients without [^18^F]FDG PET images, a uniform clonogenic cell density was assumed, yielding TCP values consistent with the rest of the cohort but without the assessment of [^18^F]FDG-derived heterogeneity. The linear calibration between [^18^F]FDG SUV and clonogenic cell density should be regarded as a feasibility construct, providing exploratory model–based estimates rather than clinical outcome surrogates. Although [^18^F]FDG uptake primarily reflects clonogenic cell density, it can also be influenced by hypoxia-driven *GLUT1* upregulation, glycolytic activity, and tumor-associated or infectious inflammation, although radiation-induced inflammation is absent in pretreatment scans. The partial overlap with [^18^F]FMISO-derived subvolumes confirms that [^18^F]FDG is not a direct surrogate for hypoxia. However, dual-tracer imaging cannot fully disentangle these effects; rather, it reduces ambiguity and strengthens the biologic modeling of radioresistance.

Although promising, dose escalation raises concerns about side effects, which are dependent on dose levels and irradiated volume (21,22). In HNSCC, common toxicities include mucositis, xerostomia, and dysphagia (23), with long-term risks (e.g., fibrosis, bone necrosis) impacting quality of life. In a phase 1 trial, Duprez et al. (24) tested adaptive dose-painting-by-numbers targeting [^18^F]FDG–avid regions, escalating to median EQD2 values of 91 Gy (high-dose clinical target volume) and 102 Gy (GTV). Mean escalation volumes were 73.1 ± 47.3 cm^3^ and 13.1 ± 13.0 cm^3^. No grade 4 or higher toxicity was reported, although mucosal ulcers occurred, particularly with higher doses. Rasmussen et al. (25) conducted a phase 1 trial with 15 HNSCC patients using [^18^F]FDG PET–guided dose painting with weekly cisplatin. Two dose-escalation strategies targeted the [^18^F]FDG PET–avid volume, including an initial uniform EQD2 prescription of 82 Gy, followed by an accelerated regimen of 34 fractions of 2.34 Gy to the [^18^F]FDG PET–avid volume. Unlike prior studies (21,22), Rasmussen et al. (25) found no correlation between boosted volume size and ulceration risk. Olteanu et al. (26) found that grade 4 ulcers occurred most often when the minimum dose received by the hottest 1.75 cm^3^ exceeded 84 Gy and EQD2 surpassed 95.5 Gy for 1.75 cm^3^. In our study, the average EQD2 in HTV was 81.0 ± 3.2 Gy, with most HTVs smaller than the threshold associated with high-grade toxicity. Despite cohort differences, our escalation levels and volumes were within or below tolerable limits reported in the literature, supporting the clinical feasibility of our approach. The recent randomized study by de Leeuw et al. (27) tested [^18^F]FDG PET–based dose painting but found no improvement in local control or progression-free survival. This highlights the limitation of [^18^F]FDG alone. In our cohort, [^18^F]FDG–avid and hypoxic regions only partially overlapped, reinforcing the rationale for multitracer strategies that may overcome shortcomings of single-tracer trials and guide future clinical validation.

In this proof-of-concept study, we focused on dose escalation to demonstrate feasibility within a biologically guided framework. Nonetheless, de-escalation is equally relevant, especially in human papillomavirus–positive and hypoxia-negative tumors. In our cohort, 36% of patients showed no detectable hypoxia, indicating that future strategies could integrate escalation to hypoxic subvolumes with de-escalation in normoxic regions to maintain tumor control while reducing radiotherapy toxicity (28).

This study utilized quantitative data from multiple PET modalities, acknowledging limitations such as partial-volume effects, tissue vasculature, and perfusion. Although these factors introduce uncertainty, the controlled trial setting and standardized protocols help mitigate their impact, even if not entirely eliminating it.

The study involved images acquired at different time points (CT_plan_, [^18^F]FDG PET/CT, and [^18^F]FMISO PET/CT), raising considerations about image registration. Rigid registration was selected over deformable registration on the basis of preliminary vector field assessments in the TV2 area, which showed minimal vector displacement (smaller or equal to the PET voxel size), well within the typical uncertainty of deformable algorithms (29). This was attributed to the close temporal proximity of acquisitions and pretreatment timing, minimizing anatomic changes. In a few cases, a larger vector displacement (<1 cm) was observed at the TV2 periphery (relatively distant from the GTV and HTV), typically in areas with CT artifacts (e.g., mandible with metal implants). [^18^F]FDG uptake in these regions varied little. For [^18^F]FMISO PET, HTVs were delineated from pO_2_ maps rigidly registered to the CT_plan_, making deformable registration unnecessary. The only VOI that could have benefited from deformation was the neck muscle VOI (used for average tracer uptake), but due to minimal displacement and large VOI size, any effect on uptake was negligible. Additionally, applying deformable fields introduces spatial interpolation, which can alter pO_2_ values and add uncertainties that are difficult to quantify. Overall, due to its intrinsic averaging nature, contour-based dose painting, which is used here, is more robust to such uncertainties compared with voxel-based methods. Recent investigations in a similar patient group have shown that the choice of CT_plan_-to-PET registration method at the pretreatment stage has a negligible impact on TCP (29). We emphasize that rigid registration, aligned on bony anatomy, was sufficient in this pretreatment setting, but deformable registration will be important for adaptive strategies once midtreatment anatomic changes are considered.

The proposed imaging-based dose-escalation method was implemented on pretreatment [^18^F]FDG and [^18^F]FMISO PET images to derive personalized biologically guided dose prescriptions. Although pretreatment imaging has shown good prognostic value in radiotherapy (30), its use for treatment individualization remains limited. Barriers include the complexity of accounting for multiple adverse factors, central to this study, and the potential for biologic changes during treatment. The proposed strategy could be extended into a broader framework incorporating longitudinal imaging to monitor early tumor responses, enabling adaptive treatment adjustments. In this cohort, additional [^18^F]FMISO PET/CT scans at weeks 3 and 5 will allow exploration of dynamic anatomic and biologic changes during treatment and their use in adaptive replanning. Such adaptive approaches are particularly relevant in HNSCC, where tumor shrinkage and reoxygenation are common, and may further improve both tumor control and OAR sparing. Indeed, prior studies have shown that [^18^F]FDG- and [^18^F]FMISO-derived tumor characteristics can distinguish responders from nonresponders (31), supporting the potential of midtreatment imaging to guide adaptive strategies. Since our method generates maps of surviving clonogenic cells, this information, combined with accumulated dose data, may support a comprehensive treatment adaptation approach. The automated treatment planning pipeline, developed for this study and described by Ureba et al. (12), was designed to enable such adaptive strategies. It achieved clinical objectives with a TCP exceeding 90% in most cases, requiring manual adjustment in only a few cases. The planning template can be further customized to specific clinical preferences and user-defined priorities for OAR sparing. Importantly, the occasional difficulties in fully meeting OAR constraints were not introduced by the biologically guided planning strategy. Similar trade-offs between target coverage and OAR sparing were also present in the clinically delivered plans, reflecting anatomic limitations inherent to head and neck cancer cases rather than a consequence of the biologically guided approach or automated optimization.

The radiobiologic core of the method assumes local changes in radiosensitivity at the cellular level over the treatment course. However, oxygenation changes detectable via functional imaging, relevant for further individualizing the treatment through biologic adaptation, are not explicitly modeled. The potential implications of these changes were anticipated at study design. Consequently, each patient underwent 3 [^18^F]FMISO PET scans, enabling future analysis of hypoxia dynamics in relation to dose and outcome. Moreover, several radiobiologic parameters (e.g., OERs, clonogenic cell density limits) were derived from the literature rather than measured on a patient-specific basis. As such, because the parameters cannot currently be measured reliably in vivo, using established values from the literature was the only feasible approach. Accordingly, the TCP values reported here, including a TCP of greater than 90%, are model-based estimates rather than clinical outcomes. In addition, the automated planning framework generates maps of surviving cells and TCP, enabling visualization of regions at higher risk of recurrence and guiding optimization. This work should be regarded as a feasibility study, intended to demonstrate the potential of dual-tracer PET for biologically guided planning and to initiate dialogue on its clinical translation. Although based on simplified assumptions, the framework provides a pragmatic starting point for refinement and prospective validation in clinical trials. Because the biologically guided dose prescriptions were not delivered, survival and toxicity outcomes are unavailable; prospective trials will be required to establish clinical relevance. Looking ahead, longitudinal PET data at weeks 3 and 5 will support investigation of adaptive strategies accounting for evolving tumor biology, and the automated planning pipeline demonstrates that biologically guided prescriptions can be integrated into existing workflows with minimal manual intervention. Future prospective clinical trials will be essential to validate the predictive value of PET-guided TCP modeling and to assess whether the dosimetric and biologic gains observed here translate to improved patient outcomes.

## CONCLUSION

The proposed biologically guided dose-escalation strategy, targeting tumor hypoxia and cell density via functional imaging, is clinically feasible and supported by dosimetric and radiobiologic evidence. This approach has the potential to advance dose adaptation strategies, allowing for tailored adjustments that can enhance tumor control and reduce radiotherapy toxicity. By moving beyond a “one-size-fits-all” model, this approach enables treatment tailored to the specific biologic properties of each tumor.

## DISCLOSURE

Financial support for this work was provided by the Cancer Research Funds of Radiumhemmet, the Swedish Cancer Society, and the Swedish Research Council (grant no. 2020-04618). Marta Lazzeroni and Iuliana Toma-Dasu report research funding from the Cancer Research Funds of Radiumhemmet. Anca Grosu reports project funding from the German Cancer Consortium (DKTK). Alexander Rühle reports a research grant from Novocure; consulting fees from Novocure and Johnson & Johnson; speaker honoraria from Novocure, Merck, and AstraZeneca; and a travel grant from Novocure (AACR 2022). Michael Mix and Nils Nicolay report research grants from the German Research Foundation (public funding) and honoraria from Novocure and Merck (outside the submitted work). Philipp Meyer reports consulting fees from Advanced Accelerator Applications Germany GmbH (paid to the author) and honoraria from Siemens Healthineers (paid to the institution). No other potential conflict of interest relevant to this article was reported.

## Notes

KEY POINTS **QUESTION:** Can combined [^18^F]FDG and [^18^F]FMISO PET guide biologically individualized radiotherapy to improve tumor control in HNSCC? **PERTINENT FINDINGS:** In this modeling study of 28 patients, biologically guided, dose-escalated treatment plans based on [^18^F]FDG-derived clonogenic cell density and [^18^F]FMISO-derived hypoxia achieved a TCP of greater than 90% while maintaining clinical feasibility of OAR sparing. **IMPLICATIONS FOR PATIENT CARE:** PET-guided, biologically individualized radiotherapy has the potential to personalize treatment by selectively escalating doses to resistant tumor regions, which may improve local control and, ultimately. patient outcomes.
